# Ortho- and Meta-monochlorinated Biphenyls Suppress Humoral Immunity and Exert Toxic Effects on Mouse Liver Cells

**DOI:** 10.32607/actanaturae.27596

**Published:** 2025

**Authors:** D. O. Egorova, S. V. Gein, N. A. Korolev, N. P. Loginova

**Affiliations:** Perm Federal Research Center, Ural Branch of the Russian Academy of Sciences (PFRC UB RAS), Perm, 614990 Russia; E.A. Vagner Perm State Medical University, Perm, 614990 Russia

**Keywords:** monochlorinated biphenyls, humoral immunity, hepatocytes, biodegradation, leukocytes

## Abstract

Widespread environmental contamination with polychlorinated biphenyls (PCBs) leads to serious health problems for humans and animals. Our main focus should be on studying the negative effects of exposure to medium- and highly chlorinated PCBs in the human body. There is limited information on the impact of low-chlorinated biphenyls containing 1–2 substituents per molecule on the functions of mammalian organs and systems. Under natural conditions, PCBs can undergo bacterial degradation; the resulting compounds belong to a group of secondary pollutants and are considered hazardous to the environment. Because of limited research, the question regarding the impact of mono-substituted chlorobiphenyl congeners, as well as the products of their biotransformation, remains open. In the presented work, the effects of *ortho*- and *meta*- substituted monochlorinated biphenyls on the functions of immune system cells and the morphofunctional state of the liver of mammals *in vivo *are revealed for the first time. PCB 1 and PCB 2 were found to suppress humoral immunity and induce a productive inflammatory response, as well as widespread protein dystrophy with necrotic foci in the liver. The products of a aerobic bacterial transformation of PCB 1 and PCB 2 were shown to not have a negative effect on the mammalian immune system but proved toxic to hepatocytes, although to a lesser extent than the original chlorobiphenyls.

## INTRODUCTION

One of the pressing issues of our time is the impact of polychlorinated biphenyls (PCBs) and their derivatives, formed in the environment under the influence of natural factors, on the human and animal organisms. Even though the Stockholm Convention prohibits the production and use of PCBs, they continue to remain in the environment and to pose a direct threat to public health [1]. The PCB group consists of 209 compounds that are different in their number of substituents and positions in the molecule. PCBs enter the human body via accumulation in food chains [2]. PCBs lead to disruption in the development of animal fetuses, are one of the causes of diabetes, cause diseases of the skin and nervous system, and provoke the emergence of cancers and genetic disorders [3, 4]. The negative effect of individual medium- and highly chlorinated PCB congeners containing more than three substituents per molecule, as well as commercial mixtures, on immunity has been demonstrated [5, 6, 7]. However, the question of the impact of low-chlorinated biphenyls on animal and human health remains open.

The main path to preventing the insertion of PCBs into food chains is by biodegrading them in the environment via the activity of the enzymatic systems of aerobic bacteria. This results in the formation of hydroxylated chlorobiphenyl derivatives and chlorobenzoic acids, which can also have negative effects when ingested by mammals [8].

In this work, the effects of *ortho*- and *meta*-substituted monochlorinated biphenyls and the products of their bacterial degradation on the parameters of adaptive immunity and the morphofunctional state of the mouse liver were investigated for the first time *in vivo*.

## EXPERIMENTAL PART

White Swiss mice of either sex weighing 18–23 g were used in this study. The animals were kept under vivarium conditions with a 12-h lighting cycle, twice-daily feeding with natural feed in an amount corresponding to daily norms, with unlimited access to water. Experiments were conducted in accordance with the recommendations and ethical standards specified in the European Convention for the Protection of Vertebrate Animals Used for Experimental and Other Scientific Purposes. Permission No. IRB00010009 was obtained from the Local Ethics Committee of the Institute of Ecology and Genetics of Microorganisms, UB RAS (Perm, Russian Federation) (Protocol No. 29 dated October 8, 2024).

*Ortho*-monochlorobiphenyl (PCB 1) and *meta*monochlorobiphenyl (PCB 2) were administered to mice orally, in corn oil, sequentially, every other day, at a dosage of 100 mg/kg. This dosage choice is based on literature sources [9]. The biodegradation products of PCB 1 and PCB 2 were administered to mice orally as an aqueous solution, every other day, at a dosage corresponding to 100 mg/kg of the initial substrate. The control groups were given corn oil and a mineral culture medium free of bacterial cells; each group contained 7–11 individuals.

The duration of the experiment was 25 days. Humoral immunity was induced on day 19 of the experiment by immunization with sheep red blood cells into the abdominal cavity, at a concentration of 108 cells in 200 μL of physiological saline. Induction of delayedtype hypersensitivity (DTH) reaction was achieved on day 24 of the experiment by inserting a resolving dose of sheep red blood cells under the skin of the left foot and an identical volume of a 0.9% NaCl solution under the skin of the right foot. On day 25, the animals were relieved from the experiment by decapitation under ether anesthesia. The humoral immune response was assessed according to the number of plaque-forming cells using localized hemolysis in a gel (Jerne plaque assay). The severity of the DTH reaction was assessed by measuring paw edema using the mass index, which was calculated using the following formula:

(*R*_exp_ – *R*_c_)/*R*_c_ × 100%,

where *R*_exp_ is the mass of the limb under experiment and *R*_c_ is the mass of the control limb.

Liver tissues were fixed in 10% neutral formalin in a phosphate buffer (pH 7.2), followed by embedding in Histomix paraffin. Histological specimens were prepared using standard histological methods. To assess the overall morphological picture in the experiment, sections were stained with hematoxylin and eosin. Evaluation and photographing were performed using an Olympus microscope (Japan) with the Imeg pro+ software package (free version).

The biodegradation products of PCB 1 and PCB 2 were obtained in experiments with washed cells of the aerobic strain *Rhodococcus *sp. FG1 (VKM Ac-3030), according to the procedure described in ref. [11]. Cultivation lasted 24 h. Quantitative analysis of chlorobiphenyls and their hydroxy derivatives was performed under GC-MS conditions [11]. The content of substances in each test sample was calculated using the internal normalization method. Qualitative analysis was performed using the NIST17 database. The contents of benzoic and chlorobenzoic acid were determined by HPLC in a culture medium freed from bacterial cells by centrifugation (9,660 g, 3 min, mini- Spin centrifuge (Eppendorf, Germany)) according to ref. [10].

Statistical analysis of the results was performed using the unpaired Student’s t-test in Microsoft Excel. The data in the tables are presented as a mean and standard error (M ± m).

## RESULTS AND DISCUSSION

As previously reported, medium- and high-chlorinated biphenyls have a suppressive effect on both humoral and cell-mediated immunity in vertebrates [5, 6, 7]. The present study demonstrated in an *in vivo* experiment that PCB 1 and PCB 2 significantly reduced the number of plaque-forming cells (PFCs) in the spleen, both in terms of relative and absolute values. However, these compounds did not have a significant effect on delayed-type hypersensitivity (DTH) (*Table 1*).

**Table 1:** The effect of ortho-monochlorobiphenyl (PCB 1) and meta-monochlorobiphenyl (PCB 2) on the number of PFCs in the spleen and the intensity of the DTH response

Substance	lgPFC/million	lgPFC/organ	DTH index, %
Corn oil (Cm)	2.25 ± 0.11	4.66 ± 0.09	20.06 ± 1.78
PCB 1	1.70 ± 0.19^*^	4.11 ± 0.20^*^	21.73 ± 2.37
PCB 2	1.69 ± 0.22^*^	4.07 ± 0.22^*^	25.59 ± 3.62

^*^*p ≤ 0.05 vs. control.

Hence, administration of PCB 1 and PCB 2 resulted in the suppression of humoral immunity, while not affecting cell-mediated immunity parameters.

It was discovered that, after microbial transformation, the products of PCB 1 and PCB 2 had no statistically significant effect on the number of plaque-forming cells in the spleen or on the intensity of the DTH response compared to the control animals that were receiving a mineral medium (Cs) used for cultivation of the microorganisms utilized for pollutant degradation (*Table 2*).

**Table 2:** The Effect of biodegradation products of orthomonochlorobiphenyl (PCB 1) and meta-monochlorobiphenyl (PCB 2) on the number of PFCs in the spleen and the intensity of the DTH response

Substance	lgPFC/million	lgPFC/organ	DTH index, %
Mineral medium (Cs)	2.04 ± 0.15	4.29 ± 0.19	30.97 ± 4.56
PCB 1 biodegradation products	2.00 ± 0.07	4.48 ± 0.11	24.63 ± 5.38
PCB 2 biodegradation products	2.02 ± 0.17	4.43 ± 0.19	19.59 ± 2.68

**Fig. 1:**
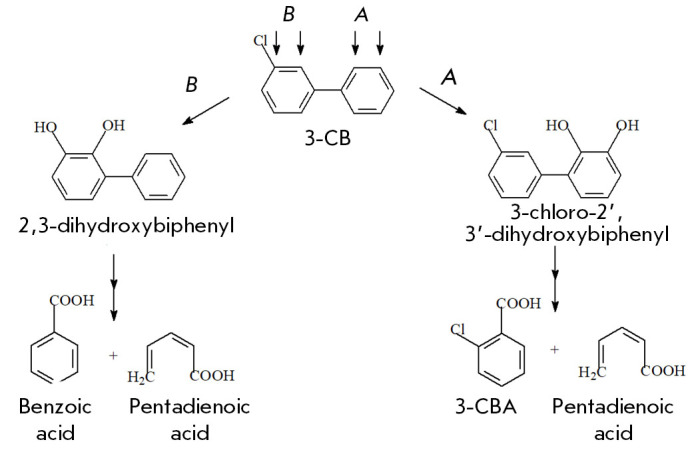
The scheme of PCB 2 oxidation by the enzymatic system of the strain *Rhodococcus *sp. FG1 and the main degradation products: (*A*) the metabolic pathway begins with oxidation of 2 and 3 carbon atoms in the unsubstituted ring of the biphenyl molecule; (*B*) the metabolic pathway begins with oxidation of 2 and 3 carbon atoms in the substituted ring of the biphenyl molecule

The GC-MS and HPLC data, as well the data in the NIST17 and KEGG databases (http://kegg.jp), appear to suggest that the strain *Rhodococcus *sp. FG1 degrades PCB 1 through the classical aerobic oxidative pathway, giving rise to 2-chlorobenzoic acid as the main compound. PCB 2 degradation results in the formation of two congeners of hydroxylated chlorobiphenyl derivatives, as well as benzoic and 3-chlorobenzoic acids (*Fig. 1*). However, in contrast to the 2,4,4’-trichlorobiphenyl metabolites [8], they do not exert a negative effect on the immune system of mice.

**Fig. 2:**
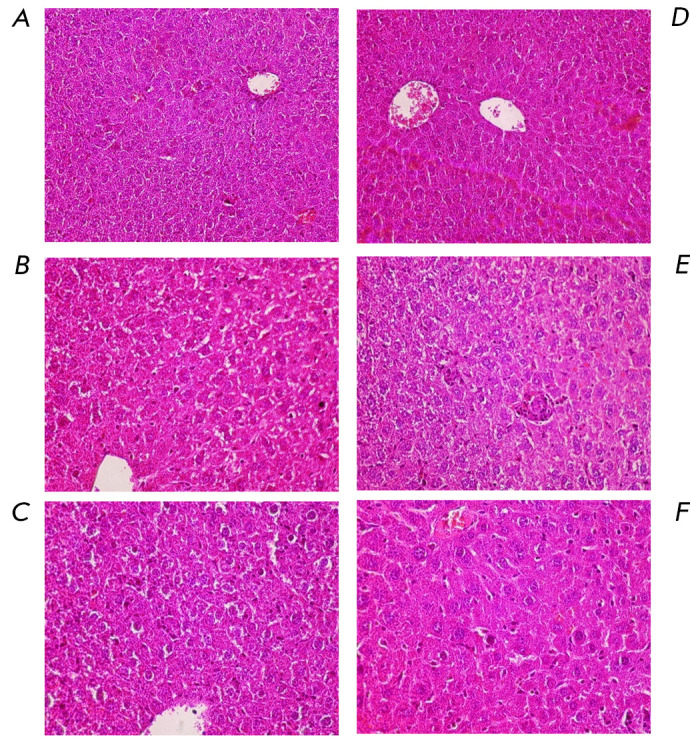
The structure of the liver of the mice in the control groups ((*A*) corn oil; (*D*) bacterial culture medium), under the influence of PCB 1 (*B*), PCB 2 (*C*), PCB 1 biodegradation products (*E*), and PCB 2 biodegradation products (*F*) by the strain *Rhodococcus *sp. FG1. Magnification: ×400. Hematoxylin and eosin staining

Histological examination of the liver revealed that the control groups presented a standard organ structure, with all the structures showing signs of functional activity (*Fig. 2A,D*). Oral administration of PCB 1 and PCB 2, as compared to the control group, resulted in a significant increase in the number of binucleated hepatocytes, as well as in that of cells with nuclei of different sizes, small foci of hepatocyte necrosis, and in a pronounced productive inflammatory reaction with signs of widespread protein dystrophy (*Fig. 2B,C*). According to ref. [11], the reaction severity was estimated at 3 points. Lugewig and Robertson [12] have noted that intraperitoneal administration of low-chlorinated biphenyls results in extensive cellular changes in liver tissues, while they found no dependence between the administered PCB congener and the severity of the resulting effect. Signs of moderate protein dystrophy of hepatocytes, moderate anisokaryosis, an increased number of binucleated hepatocytes in the central regions of the hepatic lobules, and a moderately productive inflammatory reaction were all observed in the liver of the animals that received bacterial degradation products of PCB 1 and PCB 2, (*Fig. 2E,F*). According to ref. [11], the reaction severity was estimated at 1.5 points. In light of all this, it appears safe to assume that the hydroxy derivatives of PCB 1 and PCB 2, and (chloro)benzoic acids, are less toxic to hepatocytes than the parent monochlorobiphenyls.

## CONCLUSIONS

This study has revealed for the first time that *ortho*and* meta*-monochlorinated biphenyls suppress humoral immunity and cause a productive inflammatory reaction in the liver, accompanied by signs of cellular dystrophy with necrotic foci. The products of bacterial degradation of the chlorobiphenyls under consideration do not exhibit an immunosuppressive effect; however, they continue to have a toxic effect on liver cells, albeit to a lesser extent.

## Abbreviations

PCBs: polychlorinated biphenyls
PCB 1: ortho-monochlorobiphenyl
PCB 2: meta-monochlorobiphenyl
PFCs: plaque-forming cells
GC-MS: gas chromatography-mass spectrometry
HPLC: high-performance liquid chromatography

## References

[1] Zhu M., Yuan Y., Yin H., Guo Z., Wei X., Qi X., Liu H., Dang Z. (2022). Sci. Total Environ..

[2] Frossard V., Vagnon C., Cottin N., Pin M., Santoul F., Naffrechoux E. (2023). Sci. Total. Environ..

[3] Lan T., Liu B., Bao W., Thorne P.S. (2023). Sci. Rep..

[4] Miletić M., Kmetič I., Kovač V., Šimić B., Petković T., Štrac D.Š., Pleadin J., Murati T. (2023). Env. Sci. Pollut. Res..

[5] Gao Y., Huang W., Jiang N., Fang J.K.H., Hu M., Shang Y., Wang Y. (2023). Mar. Environ. Res..

[6] Duffy J.E., Carlson E., Li Y., Prophete C., Zelikoff J.T. (2002). Mar. Environ. Res..

[7] Hammond J.A., Hall A.J., Dyrynda E.A. (2005). Aquat. Toxicol..

[8] Rengelshausen J., Randerath I., Schettgen T., Esser A., Kaifie A., Lang J., Kraus T., Ziegler P. (2023). Arch. Toxicol..

[9] (2012). Guidelines for Preclinical Studies of Medicinal Products. Part 1. M.: Grif & K, 2012. 944 p..

[10] Egorova D.O., Gorbunova T.I., Pervova M.G., Kir’yanova T.D., Demakov V.A., Saloutin V.I., Chupakhin O.N. (2020). J. Haz. Mat..

[11] Senkova A.V., Savin I.A., Chernopovskaya E.L., Davydova A.S., Meshchaninova M.I., Bishani A., Vorobyeva M.A., Zenkova M.A. (2024). Acta Naturae..

[12] Ludewig G., Robertson L.W. (2013). Cancer Lett..

